# Natural resistance to meglumine antimoniate is associated with treatment failure in cutaneous leishmaniasis caused by *Leishmania* (*Viannia*) *panamensis*

**DOI:** 10.1371/journal.pntd.0012156

**Published:** 2024-05-06

**Authors:** Olga Lucía Fernández, Mariana Rosales-Chilama, Andrea Sánchez-Hidalgo, Paola Gómez, David Esteban Rebellón-Sánchez, Ivo B. Regli, Míriam Díaz-Varela, Fabienne Tacchini-Cottier, Nancy Gore Saravia

**Affiliations:** 1 Centro Internacional de Entrenamiento e Investigaciones Médicas (CIDEIM), Cali, Colombia; 2 Universidad Icesi, Cali, Colombia; 3 Fundación Valle del Lili, Centro de Investigaciones Clínicas (CIC), Cali, Colombia; 4 Department of Immunobiology, University of Lausanne, Epalinges, Switzerland; Wadsworth Center, UNITED STATES

## Abstract

The multifactorial basis of therapeutic response can obscure the relation between antimicrobial drug susceptibility and clinical outcome. To discern the relationship between parasite susceptibility to meglumine antimoniate (SbV) and therapeutic outcome of cutaneous leishmaniasis, risk factors for treatment failure were considered in evaluating this relationship in ninety-one cutaneous leishmaniasis patients and corresponding clinical strains of *Leishmania* (*Viannia*) *panamensis*. Parasite susceptibility to 32 μg SbV/mL (plasma C_max_) was evaluated in primary human macrophages, PBMCs, and U937 macrophages. Early parasitological response to treatment was determined in lesions of a subgroup of patients, and pathogenicity of Sb-resistant and sensitive clinical strains was compared in BALB/c mice. Parasite survival in cell models and patient lesions was determined by qRT-PCR of *Leishmania* 7SLRNA transcript. Parasite loads in BALB/c mice were quantified by limiting dilution analysis. The disparate Sb-susceptibility of parasite subpopulations distinguished by isoenzyme profiles (zymodemes) was manifest in all cell models. Notably, Sb-resistance defined by parasite survival, was most effectively discerned in U937 macrophages compared with primary human host cells, significantly higher among strains from patients who failed treatment than cured and, significantly associated with treatment failure. Each unit increase in transformed survival rate corresponded to a 10.6-fold rise in the odds of treatment failure. Furthermore, treatment failure was significantly associated with naturally Sb-resistant zymodeme 2.3 strains, which also produced larger lesions and parasite burdens in BALB/c mice than Sb-sensitive zymodeme 2.2 strains. The confounding effect of host risk factors for treatment failure in discerning this association was evidenced in comparing strains from patients with and without the defined risk factors for treatment failure. These results establish the association of natural resistance to meglumine antimoniate with treatment failure, the importance of host risk factors in evaluating drug susceptibility and treatment outcome, and the clinical and epidemiological relevance of natural Sb-resistance in *L*. (*V*.) *panamensis* subpopulations.

## Author summary

The relevance of drug susceptibility of clinical strains *of Leishmania* measured in culture to the outcome of treatment is uncertain. We evaluated the relation between susceptibility of *Leishmania* isolated from cutaneous leishmaniasis patients to the widely used anti-leishmanial drug, meglumine antimoniate, and the outcome of treatment of the corresponding patients. We considered patient factors associated with treatment failure, such as age, adherence to treatment and concurrent medical conditions, in determining whether drug resistance among parasites isolated from these patients was linked to treatment failure. We found that drug-resistant *Leishmania* predominantly belong to a widely prevalent subpopulation that is biochemically distinguishable and more pathogenic in mice; and, that treatment failure was significantly more frequent (63%) among patients infected with these Sb-resistant *Leishmania* than patients infected with drug sensitive parasites (30%). Furthermore, tolerance/resistance to antimony was significantly higher among strains isolated from patients who failed treatment than those cured following treatment. The relationship between drug resistance and treatment failure was not perceived when only patients having the defined risk factors for failure were evaluated separately, evidencing the confounding effect of host factors and the importance of considering these when assessing the relevance of parasite drug resistance in the response to treatment.

## Introduction

Treatment failure is frequent for cutaneous leishmaniasis (CL) in Latin America, and estimated to be of the order of 24% overall, based on a systematic review [1]. Randomized clinical trials conducted in Colombia and other countries of Latin America have reported failure rates of 28% to 47% for meglumine antimoniate, the anti-leishmanial most widely used for the treatment of CL [2–4]. The reported variation in the clinical response to antimonial drugs in different geographical contexts has long suggested disparity in susceptibility to this first line treatment among the diverse *Leishmania* species causing CL in the Americas [2,5–8].

*In vitro* evaluation of susceptibility to anti-leishmanial drugs has provided evidence of differences in drug susceptibility among *Leishmania* species, and intra-species variation [9,10]. *L*. (*V*.) *panamensis* circulating within the Pacific Coast Region of Colombia predominantly pertains to two subpopulations discriminated by isoenzyme profiles defined as zymodemes [11]. A previously reported large scale analysis of drug susceptibility of clinical strains of *L*. (*V*.) *panamensis* isolated between 1980 and 2020 and zymodeme classification revealed that zymodeme 2.2 strains were sensitive to meglumine antimoniate *in vitro*, whereas strains pertaining to zymodeme 2.3 were resistant [9]. The demonstrated stability of this disparity in susceptibility over several decades within the expansive Pacific coast region of Colombia is consistent with natural resistance.

Despite the capacity of the anti-leishmanial drugs to kill the target parasite within the host cell, the relationship and clinical relevance of *in vitro* assessment of drug susceptibility of *Leishmania* and outcome of treatment is uncertain for all forms of human leishmaniasis [12–15]. The diversity of *in vitro* models and variability of published results of *in vitro* susceptibility testing as well as the multifactorial basis of the outcome of treatment, confound attempts to correlate drug susceptibility with clinical response to treatment. This knowledge gap impedes the consideration of drug resistance in treatment decisions or policy and the optimal use of anti-leishmanial drugs to reduce morbidity attributable to infection, and to chemotherapy.

Parasite survival and the persistence of infection following treatment and resolution of lesions is common, having been demonstrated in healthy tissues at diverse and distant body sites as well as lesion scars in a high proportion of patients [16–19]. Consequently, clinical response to treatment is not contingent upon parasite elimination. Evaluation of drug susceptibility of strains isolated pretreatment and at reactivation following therapeutic cure, revealed acquired resistance to pentavalent antimonial drug in some cases as well as primary resistance, substantiating the participation of antimonial drug resistance in reactivation in 40% of this group of recurrent cases [20]. Nevertheless, 60% of the cases of reactivation in the study population involved drug-sensitive infections, underscoring the multifactorial basis of therapeutic response in CL as well as infections caused by other microbial pathogens.

The goal of the reported research was to determine whether resistance of clinical strains of *L*. *(V*.*) panamensis* to antimonial drug as measured in cultured intracellular amastigotes, is associated with increased failure of treatment in patients with CL. To accomplish this, we have examined the interplay of parasite subpopulations, host cell models, and the influence of defined, well-documented patient risk factors for treatment failure [2,21–24] in the relation between parasite drug susceptibility and clinical outcome of treatment. The results establish the association of *in vitro/ex vivo* resistance of clinical strains of *L*. (*V*.) *panamensis* to meglumine antimoniate with failure of treatment of the corresponding patient with antimonial drug; demonstrate the confounding potential of patient risk factors for treatment failure on this association; and substantiate the clinical and epidemiological consequences of natural antimony resistance and distinct pathogenicity of *L*. (*V*.) *panamensis* subpopulations.

## Methods

### Ethics statement

The study was approved and monitored by the independent Institutional Review Board of the Centro Internacional de Entrenamiento e Investigaciones Médicas (CIDEIM) for research involving human subjects in accordance with national and international guidelines for Good Clinical Practice (Approval CEIH code: 1274). Voluntary, informed, signed consent was provided by each participant, which included retrospectively and prospectively enrolled patients, and healthy donors. Experimental protocols conducted in BALB/c mice were approved by the Veterinary Office of the Canton of Vaud, Switzerland (Authorization 3616a to FTC) and performed in compliance with cantonal and Swiss federal law as well as the principles of the declaration of Basel for animal protection.

### Research strategy

**Fig 1:**
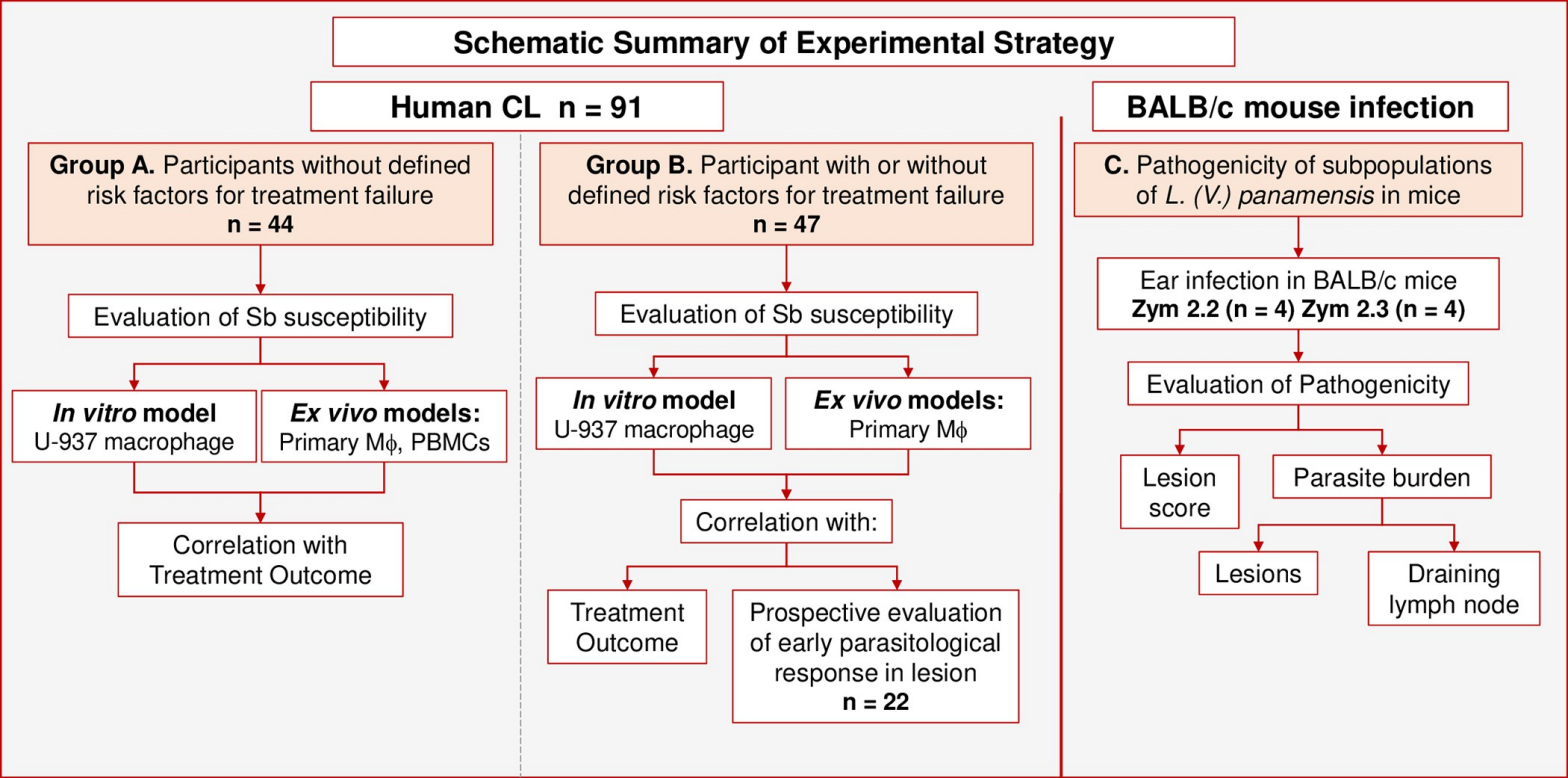
Schematic summary of experimental strategy in Human CL and Experimental infection in BALB/c Mice. **Human CL** (**Group A**): Sb-susceptibility of clinical strains of *L*. (*V*.) *panamensis* isolated from patient participants without the defined host-related risk factors for treatment failure as described in Methods and summarized in Table 1, was determined *in vitro* using the U937 cell model and *ex vivo* in monocyte derived macrophages and PBMCs. **Human CL** (**Group B**): Sb susceptibility was determined *in vitro* and *ex vivo* in a separate group of participants that included 19/47 patients having at least one of the defined risk factors for treatment failure. Sb susceptibility results in the different cell models were correlated with the early parasitological response to treatment with meglumine antimoniate in lesions of a subgroup of CL patients. **(C) BALB/c Mouse Infection:** Pathogenicity of subpopulations of *L*. (*V*.) *panamensis* belonging to zymodemes (Zym) 2.3 and 2.2, associated respectively, with natural resistance and sensitivity to antimony, was evaluated in the experimental BALB/c mouse model. Pathogenicity of infection was determined based on lesion score and parasite burden. All clinical strains included in the study were screened for LRV and found to be negative. Sb: antimony; Mϕ: Macrophages; Zym: Zymodeme. CL: cutaneous leishmaniasis.

To determine the relationship between antimonial drug susceptibility of *L*. (*V*.) *panamensis* subpopulations, and clinical response to treatment of CL, we concomitantly evaluated the susceptibility to meglumine antimoniate of clinical strains, *ex vivo* in primary human host cells consisting of monocyte-derived macrophages and peripheral blood mononuclear cells (PBMCs) from healthy donors, and in the U937 human histiocytic cell line routinely used in our laboratory to evaluate susceptibility to anti-leishmanial drugs [25]. Evaluation of drug susceptibility in different cell models sought to determine whether susceptibility in primary human host cells would approximate the response *in vivo* and hence more consistently correlate with clinical response to treatment. Parasite survival following drug exposure in U937 macrophages and primary macrophages was then correlated with the clinical outcome of treatment of 44 patients without the defined risk factors for treatment failure constituting potential confounders of the relationship between drug susceptibility and therapeutic response, group A (Fig 1A), and 47 patients enrolled irrespective of presence of these factors, group B (Fig 1B). Early parasitological response was evaluated *in situ* of lesions in a subgroup of 22 patients in group B. To investigate the interrelation of antimonial drug, parasite drug susceptibility and host cell response in the assessment of drug susceptibility and therapeutic outcome, the inclusion of study participants based on eligibility criteria was conducted both retrospectively among participants in previous clinical studies including randomized trials, and prospectively by active case finding and follow-up. All patients were diagnosed, treated and monitored according to national guidelines, with meglumine antimoniate provided by the Ministry of Health of Colombia as described in the Study Population subsection.

Further, as parasite characteristics may affect the immune and other host responses to infection and thereby the response to therapy, we experimentally evaluated the pathogenicity of clinical strains of *L*. (*V*.) *panamensis* subpopulations that are naturally resistant and sensitive to meglumine antimoniate in the BALB/c mouse model (Fig 1C).

### Study population

Patients: A total of ninety-one patients with parasitologically confirmed CL caused by *L*. (*V*.) *panamensis* having documented outcome of treatment with meglumine antimoniate, and from whom the causal parasite was isolated and identified as *L*. (*V*.*) panamensis*, were included in the study. Participants fulfilled the following inclusion criteria: voluntary participation in the study, signed informed consent/assent, and documented treatment outcome with systemic (intramuscularly administered) Glucantime. Exclusion criteria for treatment with meglumine antimoniate in clinical studies and in the management of CL in our center include co-morbidities associated with diminished immune competence or altered pharmacokinetic/pharmacodynamics: HIV/AIDS, advanced or systemic neoplasms, immunosuppressive therapy, systemic corticosteroids, immunomodulatory treatments, and antineoplastic drugs. Further, patients having conditions that constitute contraindications for administration of antimonial drugs: kidney disease, liver disease, and arrhythmias (QTc interval prolongation), and pregnancy or current breastfeeding, are not eligible for treatment with this drug and therefore were not included in this or other studies involving antimonial drug.

The study design and criteria for patient enrollment also took into consideration factors that have been generally recognized and documented to negatively influence the response to treatment of cutaneous leishmaniasis [2,21–24], including duration of lesions at the time of diagnosis, age differences in the pharmacokinetics of antimonial drugs, i.e. in young children and older adults, non-adherence to treatment, and poor drug access to lesions on cartilaginous structures of the ear. For this investigation, these factors were operationally defined as potential confounders of the relationship of the results of laboratory assessment of drug susceptibility of *Leishmania* strains isolated from patients and clinical response to treatment. Therefore, inclusion criteria for participants in Study Group A sought to obviate these confounders based on the following: time from lesion onset ≥ 1 month and < 6 months; age ≥ 8 and < 65 years; adherence to standard treatment ≥ 80%; location of cutaneous lesions at any body site except the ear. These criteria to control operationally defined confounders of the relationship between parasite drug susceptibility and clinical outcome of treatment were not applied to participants in Study Group B, who were enrolled irrespective of the defined risk factors for treatment failure.

Most participants (97%) resided in or acquired their infection in the Pacific coast region of Colombia. The demographic and clinical characteristics of the participants, and zymodemes of the corresponding clinical strains of *L*. (*V*.) *panamensis* isolated from the two patient groups included in the research strategy are summarized in Table 1. All patients received supervised standard-of-care treatment with meglumine antimoniate (Glucantime, via intramuscular injection of 20 mg SbV/kg/day for 20 days) in accordance with national guidelines (Lineamientos para la atención clínica integral de leishmaniasis en Colombia, 2018 [26] and Versión 4. 2023 [27]), and completed clinical follow-up at the end of treatment (20 days) and between 13 and 26 weeks after initiation of treatment, the time at which therapeutic outcome was determined. Cure was defined as the re-epithelialization of all cutaneous lesions and flattening of the active edge of the ulcer and absence of inflammatory signs. Therapeutic failure was defined as incomplete re-epithelialization and/or the presence of inflammation manifest as swollen, raised borders, or redness of the lesion between 13 and 26 weeks after initiation of treatment; relapse (reactivation of the lesions after initial cure), or the appearance of new lesions during follow-up.

**Table 1:** Sociodemographic, clinical and parasitologic characteristics of the study population.

Sociodemographic and Clinical Characteristics			
Sociodemographic Characteristics	Group A	Group B	Total
Total participants, No.	44	47	91
Age, median (range)	29 (9–54)	25 (2–57)	26 (2–57)
Male sex, No. (%)	33 (75%)	34 (72%)	67 (74%)
Ethnicity, No. (%)			
Afro-Colombian	29 (66%)	32 (68%)	61 (67%)
Indigenous	5 (11%)	4 (9%)	9 (10%)
Mestizo	10 (23%)	11 (23%)	21 (23%)
**Clinical Characteristics**			
Treatment Outcome (Meglumine antimoniate)—No. (%)			
Cure	25 (57%)	27 (57%)	52 (57%)
Failure	19 (43%)	20 (43%)	39 (43%)
Participants having one or more risk factors for treatment response, No. (%)		19 (40%)	19 (21%)
Patients with defined risk factors for treatment failure, No. (%):			
Age < 8 years	0	6 (12%)	6 (6%)
Age ≥ 65 years	0	0	0
Time of evolution of oldest lesion (weeks): < 4 and > 48 weeks	0	10 (20%)	10 (11%)
Treatment adherence less than 80%	0	5 (10%)	5 (5%)
Ear lesion	0	1 (2%)	1 (1%)
Co-morbidities	0	0	0
*L*. (*V*.) *panamensis* Zymodemes Identified, No. (%)			
*2*.*1*	2 (4%)	10 (21%)	12 (13%)
*2*.*2*	24 (55%)	17 (36%)	41 (45%)
*2*.*3*	18 (41%)	17 (36%)	35 (38%)
*2*.*4*	0 (0%)	3 (6%)	3 (3%)

Healthy donors: Seventeen healthy male and female donors, between 18 and 60 years of age and residence in a non-endemic area participated in this study. To limit host variability in the *ex vivo* assays, the number of healthy donors (without prior history of cutaneous leishmaniasis) was minimized, with each donor providing multiple but no more than three blood samples/year, assuring a minimum of two months between samples.

### *Leishmania* strains and lines

*Leishmania* (*Viannia*) *panamensis* strains were isolated at diagnosis from patients with cutaneous lesions by medical personnel in CIDEIM referral centers for cutaneous leishmaniasis in Cali and Tumaco, and cryopreserved in liquid nitrogen in the CIDEIM biobank. Species identification was achieved by indirect immunofluorescence using species-specific monoclonal antibodies [28] and subpopulations discriminated using isoenzyme electrophoresis based on five enzymes (6PGDH, G6PDH, MPI, NH and SOD) that distinguish the zymodemes of *L*. (*V*.) *panamensis* as previously described [11]. All clinical strains were screened for the presence of the *Leishmania* RNA virus (LRV) as described previously [29], and found to be uniformly negative. *In vitro* drug susceptibility of intracellular amastigotes was evaluated within four passages of recovery from liquid nitrogen. Prior to conducting drug susceptibility assays in the different cell models of infection (described below), the species identity was confirmed [28], and discrimination of drug susceptibility in the U937 macrophage model, was evaluated using the established CIDEIM protocol with addition of antimonial drug at 24 and 72 h after infection during a total period of culture of 96 h [25], for all strains included in this study. The antimony sensitive *L*. (*V*.) *panamensis* strain (MHOM/COL/86/1166) [25], and the antimony resistant line (MHOM/COL/86/1166-1000.1) [30] derived from this strain were employed as internal controls for susceptibility assays.

### PBMC isolation, and storage

Blood samples of 200 mL were obtained from healthy donors in sterile heparinized tubes. PBMCs were collected by centrifugation over Ficoll-Histopaque (Sigma-Aldrich), according to product instructions. Half of the isolated PBMCs were employed for differentiation of macrophages from monocytes and the other half was cryopreserved in liquid nitrogen after freezing by slow cooling at approximately 1°C/minute using a Nalgene Freezing Container (Thermo Fisher Scientific). Differentiation of peripheral blood monocytes to macrophages was achieved by culture over 7 days, at which time PBMCs recovered from liquid nitrogen by rapidly thawing at 37°C and having ≥ 90% viability [31] were evaluated in parallel with monocyte-derived macrophages that were obtained from PBMCs of the same donor.

### PBMC culture

PBMCs were cultured at a final concentration of 5 x 10^5^ monocytes/well in RPMI-1640 (22400- Gibco, USA) medium supplemented with 10% heat inactivated FBS (10082; Gibco, USA), and dispensed as 500 μL aliquots into 24-well plates (Corning Falcon ref: CLS353047, USA) immediately prior to infection with *Leishmania* (as described below: Infection and Drug exposure).

### Macrophage cell culture and differentiation

The human promonocytic cell line U937 (ATCC CRL-1593.2) and monocyte-derived primary human macrophages from healthy volunteer donors were utilized in the reported experiments.

#### U937 macrophages (*in vitro* assay)

U937 cells were differentiated to macrophages as previously described [25]. Cells were cultured at a concentration of 1.5 x 10^5^ cells/well in 24-well culture plates containing sterile round 12-mm glass coverslips (Fisher Scientific) for microscopic readout, and at 5.0 x 10^5^ cells/well in 24-well plates for evaluation of parasite survival by qRT-PCR of *Leishmania* 7SLRNA transcripts. The results obtained, demonstrated that susceptibility was concordant between the macrophage concentration adjusted to 1.5 x 10^5^ cells/well and 5 x 10^5^ cells/well in 24-well plates, both for the drug sensitive laboratory line (% parasite survival at 32 μg/mL SbV: 20.6% and 21%, respectively) and the Sb-resistant line (% parasite survival at 32 μg/mL SbV: 93.7% and 87.7%, respectively).

#### Primary human macrophages (*ex vivo* assay)

Human macrophages were differentiated from monocytes in PBMCs from healthy donors. Monocytes were isolated using anti-human CD14 MicroBeads (MACS Miltenyi Biotec CD14 MicroBeads UltraPure human), as per manufacturer protocol. Differentiation of macrophages was achieved by adherence of monocytes to 24-well plates in serum-free RPMI for 2 h at 5 x 10^5^ cells/well, followed by culture during 7 days in RPMI supplemented with 20% heat inactivated FBS at 37°C and 5% CO_2_. Infection was quantified by qRT-PCR of *Leishmania* 7SLRNA transcripts [16,32,33].

### *In vitro* and *ex vivo* assays for drug susceptibility of intracellular amastigotes

#### Infection and drug exposure

Differentiated U937 cells, primary monocyte-derived macrophages and PBMCs were exposed to infection with promastigotes previously opsonized with 10% AB positive human serum at a ratio of 5 parasites per macrophage or monocyte (PBMCs). Infected macrophages were washed once with sterile phosphate-buffered saline to remove free parasites. Subsequently, both macrophages and PBMCs were incubated for 24 h at 34°C in 5% CO_2_ in RPMI-1640 medium containing 10% heat-inactivated FBS. To evaluate parasite drug susceptibility in macrophages, medium was replaced with complete RPMI containing 32 μg SbV/mL as additive-free meglumine antimoniate (Walter Reed 214975AK; lot no. BLO918690-278-1A1W601; antimony analysis, 25% to 26.5%, by weight) and incubated for 72 h. For the evaluation of drug susceptibility in PBMCs, complete RPMI containing drug, was added without removing supernatant to achieve a final concentration of 32 μg SbV/mL. Infected cells in complete medium without drug served as controls.

#### Evaluation of intracellular survival of clinical strains by microscopy to confirm drug susceptibility using the established U937 macrophage model

Parasite survival was assessed, as previously described [25]. Glass coverslips with infected cells were fixed with methanol, stained with 3% Giemsa (Sigma-Aldrich, USA), and then evaluated in a blinded methodology by one of three experienced microscopists. Six replicates of infected cells exposed to drug and infected control macrophages without drug were evaluated for each clinical strain. The number of intracellular amastigotes per cell was determined for 100 macrophages per replica.

#### Evaluation of intracellular survival of clinical strains by qRT-PCR

The viability of clinical strains of *Leishmania* in the *in vitro* and *ex vivo* models was evaluated by amplifying transcripts of the 7SLRNA gene of *Leishmania* as previously described [16,32,33]. Three replicates of infected cells (PBMCs, U937 and primary macrophages) exposed to drug and infected control were preserved in TRIzol Reagent (Thermo Fisher Scientific) and stored at -80°C until processed. RNA was extracted according to Invitrogen TRizol manufacturer recommendations. High-capacity cDNA reverse transcription kits (Applied Biosystems) were used for cDNA synthesis. cDNA was used for qRT-PCR of *Leishmania* 7SLRNA transcripts to quantify parasite burden [16]. The single copy gene coding for the human Peptidylprolyl Isomerase B protein (PPIB) [34] was amplified for quantitation of nucleated human cells. The number of viable parasites was calculated by extrapolation using a standard curve generated and normalized to the number of human nucleated cells using PPIB amplification. Standard curves for quantitation of parasites and human nucleated cells were constructed by ten-fold serial dilution of cDNA products obtained from 1 x 10^7^ *L*. (*V*.) *panamensis* promastigotes and from the human U-937 promonocytic cell line (1 x 10^7^ cells), respectively. Detection of amplification products was performed using SYBR Green Master Mix (Applied Biosystems) on a BioRad CFX-96 platform. The specificity of qRT-PCR products was determined by melting curve analysis. Quantification of parasites by qRT-PCR was conducted blindly.

For *in vitro/ex vivo* assays, drug susceptibility was expressed as percent parasite survival, determined by dividing the parasite burden of infected cells exposed to the drug by that of infected cells without drug and multiplying by 100.

#### Standardization of primary host cell models for evaluation of susceptibility to meglumine antimoniate using qRT-PCR of *Leishmania* 7SLRNA

In order to evaluate drug susceptibility *ex vivo* in primary monocyte derived macrophages and PBMCs and avoid loss of infected and uninfected non-adherent cells, meglumine antimoniate was added at a concentration of 32 μg/ml once at 24 h post-infection followed by incubation for 72 h. In the previously established and described U937 model for assessment of drug susceptibility, culture medium was removed and replenished together with drug at 24 and 72 h [9]. The disparity in susceptibility to antimony of zymodeme 2.2 and 2.3 subpopulations of *L*. (*V*.) *panamensis* was maintained whether infected U937 macrophages were exposed to one or two additions of 32 μg SbV/mL over 72 h (S1A and S1B Fig). Quantification of viable parasites using qRT-PCR of the *Leishmania* 7SLRNA transcript was implemented both to enable quantitative readout of parasite survival in primary host cell populations in which monocyte derived macrophages may detach during culture and lymphocytes obscure microscopic evaluation, and to improve the efficiency of readout of viability across all cell models. The analysis of antimonial drug susceptibility of *L*. (*V*.) *panamensis* subpopulations using qRT-PCR of 7SLRNA and a single dose of meglumine antimoniate at 24 h, reproduced the results obtained by microscopic evaluation of naturally Sb-resistant and sensitive strains (S1C Fig). Correlation of parasite survival determined by qRT-PCR of the *Leishmania* 7SLRNA transcript and microscopic readout was evaluated in 40 clinical strains based on susceptibility data for the single addition of drug. A high (*r* = 0.8097) and statistically significant (*P = 0*.*0001*) correlation between susceptibility determined by the two methods was substantiated (S1D Fig). Analyses of clinical strains isolated during the decade of 1980–1990 as an internal reference for the evaluation of susceptibility to meglumine antimoniate using the adapted protocol for evaluation in primary cells, corroborated the fidelity of the methodology and the stability and persistence of the disparate antimony susceptibility of *L*. (*V*.) *panamensis* subpopulations discriminated by isoenzyme polymorphisms (S1E Fig) [11].

### *In vivo* parasitological response to treatment with meglumine antimoniate

To evaluate the early *in vivo* parasitological response to treatment, samples of cutaneous lesions were obtained from a subgroup of 22 prospectively enrolled patients using sterile swabs (Puritan: two Ref 253318H; and one Ref 2515061PF) by gently rubbing two swabs, one each of the aforementioned references, and separately, an additional swab of reference 253318H over the ulcerated surface of the lesion. Lesion samples were obtained from these patients before treatment, at day two or three, and day eight after initiation of treatment. All swab samples were preserved in TRIzol Reagent (Thermo Fisher Scientific, USA) and stored at -80°C until performance of quantitative qRT-PCR of 7SLRNA. Parasitological response to treatment was expressed as the reduction of parasite burden in the lesion at day 2/3 and 8 after initiation of treatment with meglumine antimoniate, compared to the parasite burden prior to treatment.

#### Mice

Female BALB/c mice were purchased from Envigo (Cambridgeshire, United Kingdom). Mice were housed in the Epalinges Center under pathogen-free conditions and used for experiments at 6 weeks of age.

### Pathogenicity of clinical strains in the experimental BALB/c mouse model

Metacyclic parasites were isolated using Percoll gradient as previously described [35]. Metacyclic parasites (1 X 10^5^) from *L*. (*V*.) *panamensis* zymodeme 2.2 or 2.3 strains were needle-injected into the ear dermis of BALB/c mice. Lesion development was monitored weekly during 6 weeks using a caliper, and lesion score was calculated as previously described [36]. A score between 0 and 8 was attributed according to inflammation, lesion size and ear integrity. Briefly, naïve ears have a score of 0, whereas the appearance of inflammation indicates a score of 0.5. When the lesion was delimited, lesion size was measured by caliper. The appearance of necrosis was scored as 6, whereas 8 defined tissue destruction.

The parasite load present in the infected ears and in the draining lymph nodes (dLNs) was analyzed by limiting dilution assay at 6 weeks of infection. Mice were euthanized and infected ears as well as dLNs were collected and processed to obtain single cell suspensions. Briefly, the dermal layers of ear tissue were separated with forceps, homogenized, and digested using 0.2 mg/mL Liberase (Roche) for 2 h at 37°C. Digested ear tissue was then filtered through 40 μm filters. Cell suspensions from the dLNs were obtained using a glass homogenizer. To estimate the number of living parasites in the infected tissues, cell suspensions were serially diluted in RPMI medium supplemented with 10% FBS, overlaid in 96-well plates containing 100 μL of rabbit blood agar and cultured for 10 days at 26°C. Presence of parasites in the wells was determined by microscopy and the number of parasites was calculated using the ESTIMFRE software as previously described [37,38].

### Statistical analysis

Analyses were performed in R version 4.1.2, STATA 16 and GraphPad Prism version 9 as follows.

#### Cell models

Comparison of the Sb susceptibility of clinical strains to meglumine antimoniate in the three host cell models (U937, primary macrophages and PBMCs) was conducted using paired statistical tests: Friedman test or the repeated measures one-way ANOVA using the Geisser-Greenhouse correction, in accordance with the distribution of data. Sb susceptibility in U937 and primary macrophage cell models was compared using the Wilcoxon matched-pairs signed-rank test, and the Mann-Whitney test was used to compare Sb susceptibility of zymodemes 2.1/2.2 and 2.3.

#### Antimony susceptibility and zymodeme in the response to treatment

Logistic regression was used to evaluate drug susceptibility and zymodeme as parasite risk factors for treatment failure. When expressed as a percentage, the parasite survival values were skewed, particularly in primary macrophages and PBMCs, and hence were fourth-root transformed to prevent high values from having excessive influence. Exploratory spline analysis to examine non-linearity by generalized additive logistic models [39] showed that the log-odds were approximately linear with the transformed susceptibility, and therefore analyzed as a continuous predictor, rather than categorized.

#### *In situ* parasitological response to treatment

Parasite burden at the lesion site of CL patients was compared between those who cured and failed antimonial treatment, and between zymodeme groups, using the Mann-Whitney test. At post-treatment time points, the parasite burden was expressed as a percentage of the pre-treatment value. Comparison of the parasite burden in the lesion based on samples obtained with the two references of swabs before or after treatment, showed no significant differences in the quantification of the parasite burden. Based on these results, the mean of the three swab samples obtained from the lesion of each patient was used to determine the parasitological response *in situ*.

#### Pathogenicity of zymodeme 2.2 and 2.3 strains in BALB/c Mice

Pathogenicity scores of zymodeme 2.2 and 2.3 strains *of L*. (*V*.) *panamensis* in BALB/c mice were compared using two-way analysis of variance (ANOVA), with the two factors being time and the specific infecting parasite strain. Parasite burden was measured at the final time point (six weeks p.i.) by limiting dilution analysis (LDA) and data were analyzed using the Mann-Whitney test.

## Results

### Natural antimony resistant and sensitive subpopulations of *L* (*V*.) *panamensis* were discernable in both *in vitro* and *ex vivo* host cell models

**Fig 2:**
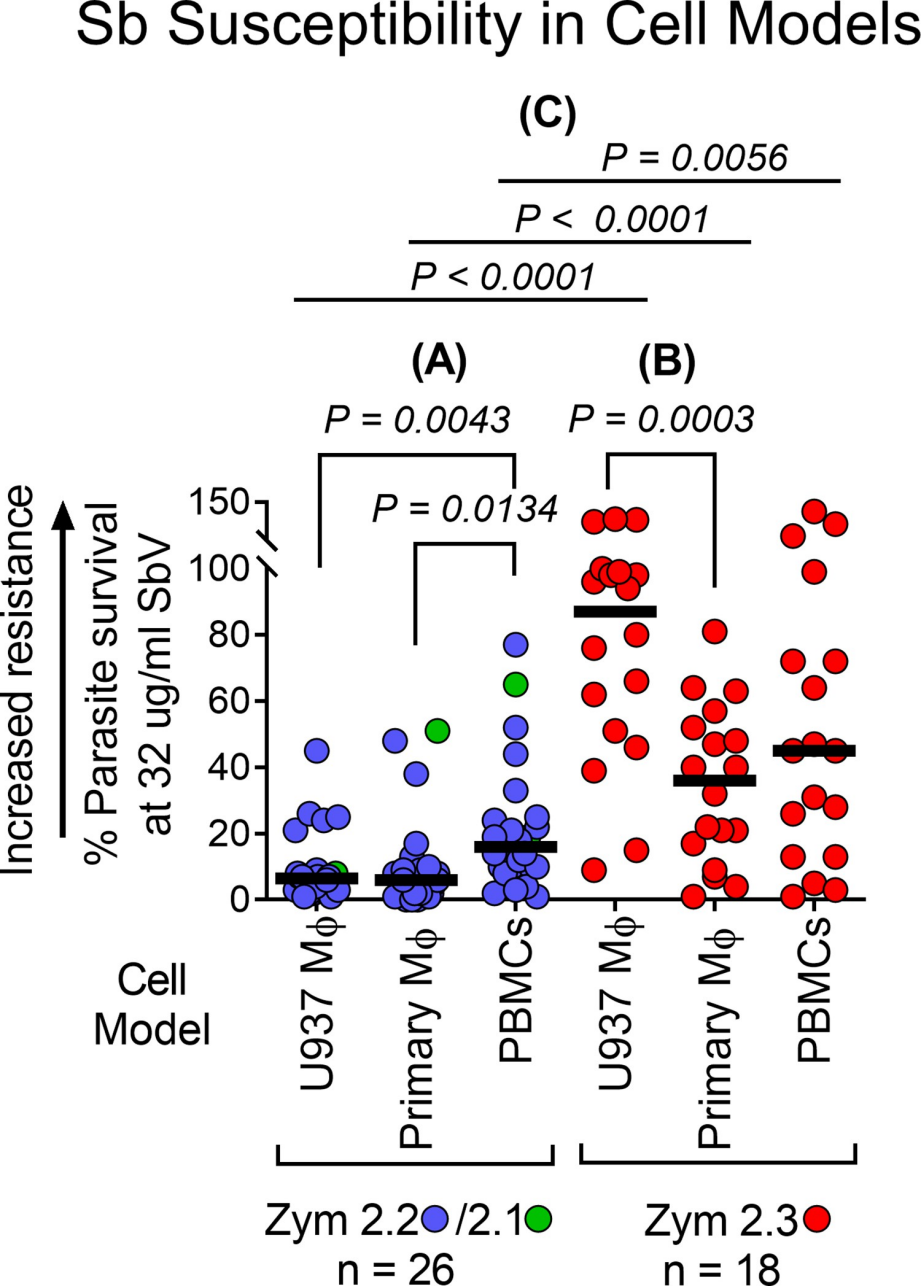
Discrimination of distinct antimony susceptibility of subpopulations of *L*. (*V*.) *panamensis* using *in vitro* and *ex vivo* cell models. **(A)** Comparison of Sb susceptibility of clinical strains belonging to zymodemes 2.1/2.2 across individual cell models (U937 macrophages, primary human macrophages, and PBMCs); **(B)** Comparison of Sb susceptibility of clinical strains belonging to zymodeme 2.3 across individual cell models **(C)** Comparison of Sb susceptibility of clinical strains of zymodeme 2.1/2.2 and zymodeme 2.3 within each cell model. Data are expressed as % parasite survival after 72 h exposure to 32 μg SbV/mL. Each data point corresponds to the mean % parasite survival determined by qRT-PCR of *Leishmania* 7SLRNA transcripts of three replicate cultures of each strain. The bar indicates median % survival of each subpopulation of clinical strains. The Mann-Whitney test was used to compare zymodemes 2.1/2.2 and 2.3. Differences among cell models, within each zymodeme, were evaluated using paired statistical tests: Friedman test (2.1/2.2 strains) or the RM one-way ANOVA (2.3 strains), according to the distribution of the corresponding data. Mϕ: Macrophages; Zym: Zymodeme.

Susceptibility to meglumine antimoniate in primary macrophages and PBMCs, which sought to approximate host cell infection *in vivo*, reproduced the distinct antimony susceptibility of *L*. (*V*.) *panamensis* subpopulations initially revealed in the U937 model. However, the variance and median percent survival of clinical strains differed among the *in vitro* and *ex vivo* cell models. Evaluation of Sb susceptibility of clinical strains from patients without known risk factors for treatment failure (Fig 1, group A) demonstrated that the subpopulation of *L*. (*V*.) *panamensis* corresponding to zymodeme 2.2, and closely related to zymodeme 2.1 [11] were sensitive to meglumine antimoniate in all cell models (Fig 2A), and that zymodeme 2.3 strains presented significantly higher percent survival indicative of Sb tolerance or resistance in all models (Fig 2B). However, discernment of resistance was most consistently achieved in U937 macrophages (median % parasite survival: Zym 2.2 and Zym 2.1 = 6.5% vs Zym 2.3 = 87%, *P < 0*.*0001*) compared with either primary macrophages (median % parasite survival: Zym 2.2 and Zym 2.1 = 6.0% vs Zym 2.3 = 36%, *P < 0*.*0001*) or PBMCs (median % parasite survival: Zym 2.2 and Zym 2.1 = 15.5% vs Zym 2.3 = 45%, *P < 0*.*0056*) (Fig 2C). Clinical strains of all zymodeme subpopulations evidenced greater variance in susceptibility in PBMCs, with marginally yet significantly higher survival of Sb-sensitive zymodeme 2.1/2.2 strains exposed to antimony in PBMC cultures compared to U937 and primary macrophages. Non-removal of supernatants and in turn extracellular parasites following infection of this non-adherent cell model may have contributed to this variance.

### Natural resistance to antimony of clinical strains of *L*. (*V*.) *panamensis* was more effectively discerned in the U937 macrophage cell line than in primary macrophages

**Fig 3:**
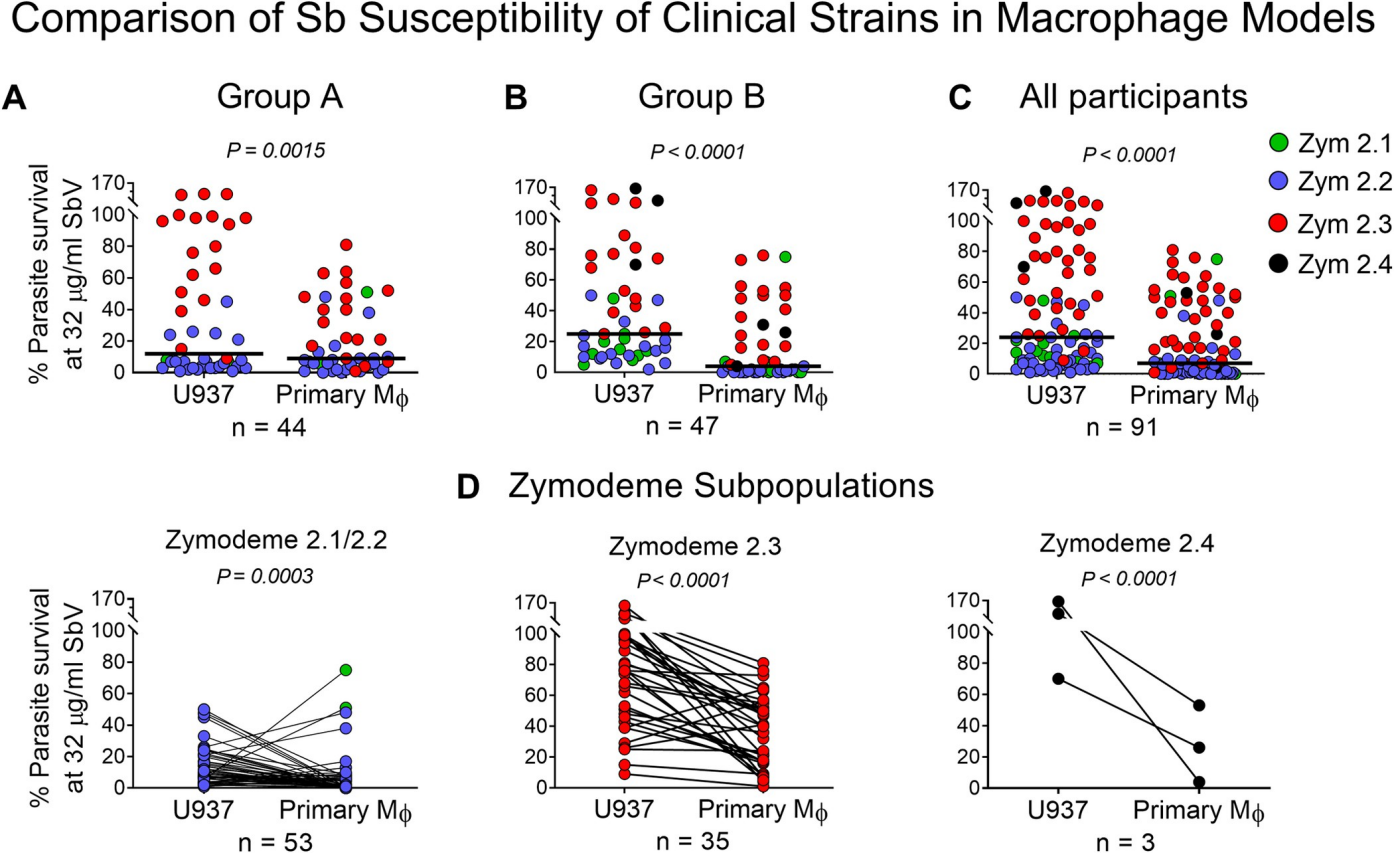
Comparison of percent survival of intracellular amastigotes of clinical strains of *L*. (*V*.) *panamensis* exposed to SbV in U937 macrophages and monocyte-derived primary human macrophages. Scatter plot of % survival of clinical strains isolated from patients: **(A)** group A, **(B)** group B, and **(C)** all participants, at 72 h exposure to SbV as meglumine antimoniate. **(D)** Paired comparison of % survival of intracellular amastigotes of individual clinical strains in U937 macrophages and primary human macrophages by zymodeme subpopulation. Statistical analysis was conducted using Wilcoxon matched-pairs signed-rank test. Sb: antimony; SbV: pentavalent antimony; Mϕ: Macrophages; Zym: Zymodeme.

Subsequent analysis of strains from patients Group A (excluding patients the defined risk factors of treatment failure) (Fig 1A), and Group B (including patients with and without known risk factors for treatment failure) (Fig 1B), in U937 and primary macrophages further demonstrated that natural tolerance or resistance to antimony, manifested as survival of intracellular amastigotes exposed to 32 μg SbV/mL, was significantly higher in the U937 model compared with primary human macrophages (Fig 3A, 3B and 3C). Conversely, increased potency, i.e. parasiticidal activity of meglumine antimoniate, was evidenced by significantly lower parasite survival in primary human macrophages, consistent with the participation of the antimicrobial capacity of primary macrophages in the anti-leishmanial effect of exposure to antimonial drug *ex vivo*. Lower parasite survival in primary macrophages was evident post-exposure to meglumine antimoniate for both sensitive (2.1 and 2.2) and naturally Sb-resistant 2.3 clinical strains, and strains identified as zymodeme 2.4 (Fig 3D).

### Failure of treatment with meglumine antimoniate was significantly associated with antimony resistance and zymodeme of *L*. (*V*.) *panamensis*

**Fig 4:**
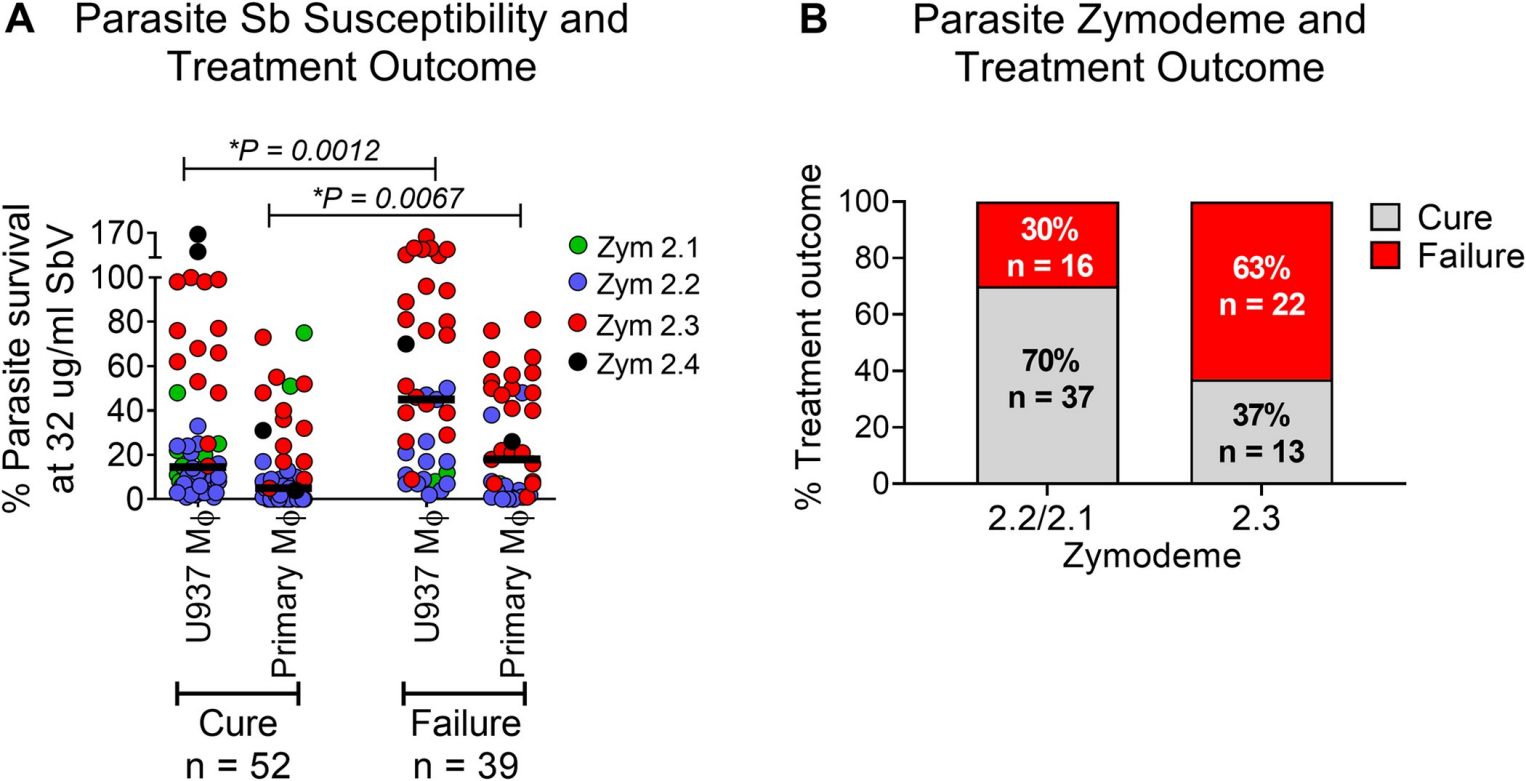
Association of parasite drug susceptibility and zymodeme with treatment failure in CL caused by *L*. (*V*.) *panamensis*. **(A)** *In vitro* and *ex vivo* survival following 72 hours exposure to 32 μg SbV/mL of clinical strains of *L*. (*V*.) *panamensis* from patients who were cured, or failed treatment with meglumine antimoniate, *The Mann-Whitney test was used to compare Sb susceptibility of strains isolated from patients who cured and failed treatment in each cell model; **(B)** Percent of CL patients infected with *L*. (*V*.) *panamensis* zymodeme 2.2/2.1 or 2.3 subpopulations who cured or failed treatment with meglumine antimoniate. Logistic regression was used to evaluate drug susceptibility (MDMs: *P = 0*.*008*; U937: *P = 0*.*004*) and zymodeme (*P = 0*.*003*) as parasite risk factors for treatment failure. Sb: antimony; SbV: pentavalent antimony; Mϕ: Macrophages; Zym: Zymodeme.

Patients infected with the subpopulations defined by zymodeme and having distinct susceptibility to meglumine antimoniate, experienced disparate clinical responses to this antileishmanial drug. Supporting the relationship between parasite susceptibility to antimonial drug and treatment outcome, intracellular parasite survival in both U937 and primary macrophages after exposure to 32 μg SbV/mL (indicative of drug tolerance or resistance), was significantly higher among strains isolated from patients who failed treatment than those who cured (Fig 4A) and, significantly associated with treatment outcome. Based on univariate logistic regression analysis each unit increase in the transformed survival rate in primary macrophages corresponded to a 10.6-fold rise in the odds of treatment failure (95% CI: 1.8–61.2; *P* = *0*.*008*). Likewise, analysis of survival in U937 macrophages confirmed this with an OR of 22.5 (95% CI: 2.8–183.6; *P = 0*.*004*). Higher survival rates of clinical strains of *Leishmania* when exposed *in vitro/ex vivo* to meglumine antimoniate indicated a heightened risk of clinical treatment failure. Further, cutaneous leishmaniasis caused by zymodeme 2.3 strains presented a strong association with treatment failure (22/35, 63%) as evidenced by an odds ratio-OR of 3.9 (95% CI: 1.6–9.6) whereas CL caused by zym 2.1 and zym 2.2 strains experienced a low proportion of treatment failure (16/53, 30%) (Fig 4B); these differences were statistically significant (*P = 0*.*003*). Utilizing a Monte Carlo simulation approach with 500 iterations, the relationship between parasite susceptibility to antimonial drugs, expressed as the median percentage of intracellular parasite survival and treatment outcomes resulted in a statistical power of 99.6% with an alpha level of 0.05 (Fig 4A). For the association between zymodeme subpopulation and treatment response, a two proportion comparison was utilized to calculate the statistical power of the study population consisting of 53 patients infected with zymodeme 2.2/2.1 strains and 35 patients infected with zymodeme 2.3 strains (Fig 4B). The statistical power achieved was 87.5%. The relationship between infection with zymodeme 2.4 strains and response to treatment was not analyzed because of the small number of patients (n = 3) that were infected with this subpopulation of *L*. (*V*.) *panamensis*.

### Defined host risk factors in the relationship between drug susceptibility and outcome of treatment

Forty percent (19/47) of patients included in Group B and 21% (19/91) of study participants overall, presented one or more of the defined risk factors for treatment failure (Table 1). The 19 patients having at least one of the defined risk factors included in the study design experienced a higher proportion of failures (68%) compared to patients who did not present any of these risk factors (36%); OR: 3.8 (95% CI: 1.3–11.3; *P = 0*.*01)*. Remarkably, among patients having at least one of the defined risk factors for treatment failure, the percent failure for patients infected with the Sb-sensitive zymodeme 2.1/2.2 strains (n = 9) and the Sb resistant zymodeme 2.3 strains (n = 9) was identical (67%). The single patient having defined risk factors and infected with a zymodeme 2.4 strain also failed treatment. On the other hand, the inclusion of the 19 patients having these risk factors for treatment failure in the analyses of Group B, or together with the 44 patients in Group A who did not present such factors, did not alter the significant association of Sb susceptibility or zymodeme with treatment outcome. After adjusting for risk factors using multivariable logistic regression, association between infection with zym 2.3 and failure, remained significant (adjusted odds ratio-aOR: 3.8; 95% CI: 1.5–9.5; *P = 0*.*005*). Similarly, the relationship between parasite survival of exposure to the plasma C_max_ of Sb and treatment outcome remained significant after adjusting for the presence of at least one risk factor: aOR: 10.10 for primary macrophages (95% CI: 1.6–62.5, *P = 0*.*001*) and aOR: 17.3 for U937 macrophages (95% CI: 2.0–148.8. *P = 0*.*009*).

To the contrary, neither the association of zymodeme nor Sb susceptibility with response to treatment was discerned in the separate analysis of the 19 patients having at least one of the defined risk factors for treatment failure. Moreover, survival of intracellular amastigotes of the corresponding clinical strains exposed to meglumine antimoniate *in vitro* was not significantly different between strains isolated from patients who cured and those who failed, both in the primary macrophage model (median % parasite survival: cure = 21% vs failure = 7%, *P = 0*.*208*) and, the U937 model (median % parasite survival: cure = 35% vs failure = 47%, *P = 0*.*424*). Hence, the ability to discern the relationship between parasite (microbiological) susceptibility to meglumine antimoniate and outcome of treatment, and the association of susceptibility to antimonial drug with zymodeme and outcome of treatment was obscured in patients presenting even one of the defined risk factors for treatment failure.

#### Lesion characteristics and outcome of treatment

The characteristics of patient lesions were evaluated for the overall study population and according to treatment outcome to identify potential bias that may have been introduced by lesion presentation in the relationship between *in vitro* drug susceptibility and *L*. (*V*.) *panamensis* subpopulations defined by isoenzyme profile (zymodeme) and the outcome of treatment with meglumine antimoniate. There was no significant difference in the number or area of lesions between patients that cured or failed treatment (S1 Table). The vast majority 82/91 (88.2%) presented ulcerated lesions. However, a significant difference (*P = 0*.*01*) in lesion presentation in relation with cure and failure emerged, with all seven patients presenting plaques, among the 11 having non-ulcerated lesions, being cured by treatment with meglumine antimoniate. Five (71%) of the corresponding strains, pertained to the Sb-sensitive zymodemes 2.1/2.2, and two (29%) with zymodeme 2.3, evidencing that susceptibility to meglumine antimoniate and the curative response to treatment in this small group of patients presenting plaque lesions concurred with the predominance of zymodemes 2.1/2.2.

### Early *in situ* reduction of parasite burden by standard of care treatment with meglumine antimoniate in relation with treatment outcome and zymodeme of infecting strain

**Fig 5:**
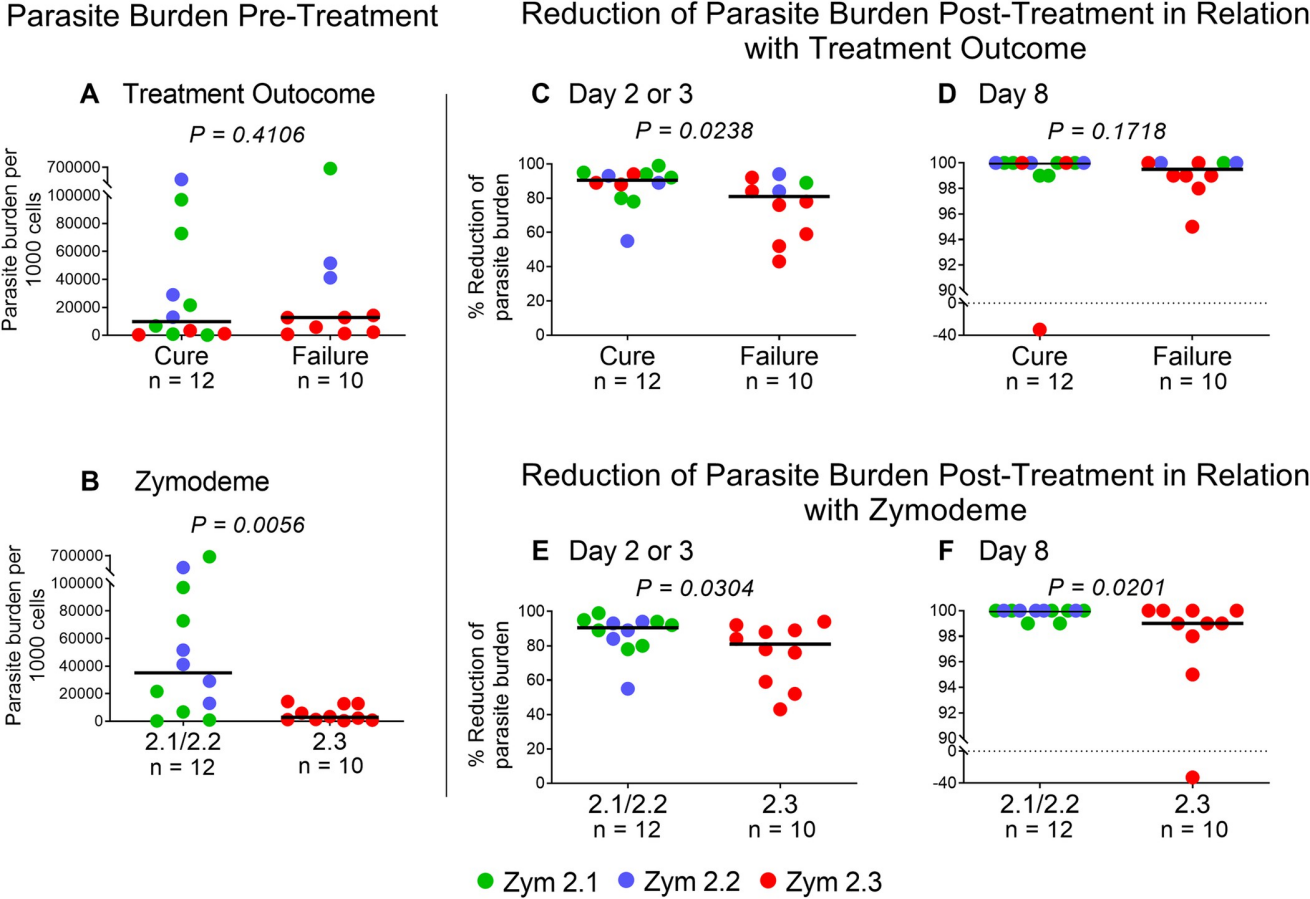
*In situ* parasitologic response to treatment with meglumine antimoniate. Pre-treatment parasite burden in lesions of patients **(A)** in relation with treatment outcome (cure or failure) and **(B)** in relation with the zymodeme subpopulation of the infecting parasite. Post-treatment reduction of parasite burden in CL lesions **(C)** on Day 2 or 3, and **(D)** Day 8 of treatment in patients who cured or failed treatment, and **(E)** in lesions on Day 2 or 3 and **(F)** Day 8 in relation with zymodeme subpopulation of the strain isolated from the corresponding patient. The Mann-Whitney test was used to compare the parasite burden among the groups by cure, failure, and zymodeme. Zym: Zymodeme.

No difference in parasite burden at diagnosis, prior to initiation of treatment with meglumine antimoniate, was detected in lesions of patients who cured compared to those who failed to respond to treatment (Fig 5A). However, the parasite burden in CL lesions at diagnosis that were caused by infection with zym 2.2/2.1 strains was significantly higher than that of lesions attributable to zym 2.3 strains (Fig 5B). Prospective *in situ* evaluation of the parasitological response to standard of care treatment with meglumine antimoniate using qRT-PCR of 7SLRNA transcripts, revealed a rapid and high percent reduction of parasite burden by day 2 or 3 after initiation of treatment in all patients. Nevertheless, at this initial time interval, a significantly lower percent reduction of parasite burden was detected in lesions of patients who failed treatment compared with patients who cured (Fig 5C). By day 8 no statistically significant difference in parasite burden was detected in lesions of patients who cured or failed to heal at 13 weeks (initial response) or 26 weeks (final response) after initiating treatment, (Fig 5D). The majority of the patients whose parasitological response in the lesion site was evaluated, and who failed to respond to treatment (7/10) were infected with zymodeme 2.3 strains of *L*. (*V*.) *panamensis*, whereas most patients (9/12) who cured, were infected with zymodeme 2.2/2.1 strains (Fig 5A). Analysis of the parasitological response stratified by infecting zymodeme subpopulation, also revealed a significantly lower percent reduction of parasite burden both at day 2 or 3, and day 8 of treatment, in lesions attributable to infection with zymodeme 2.3 strains (Fig 5E and 5F), corroborating the evidence of the participation of natural antimonial drug susceptibility in therapeutic response.

### Pathogenicity of *L*. (*V*.) *panamensis* strains of zymodeme 2.2 and 2.3 differs in the experimental BALB/c mouse model

**Fig 6:**
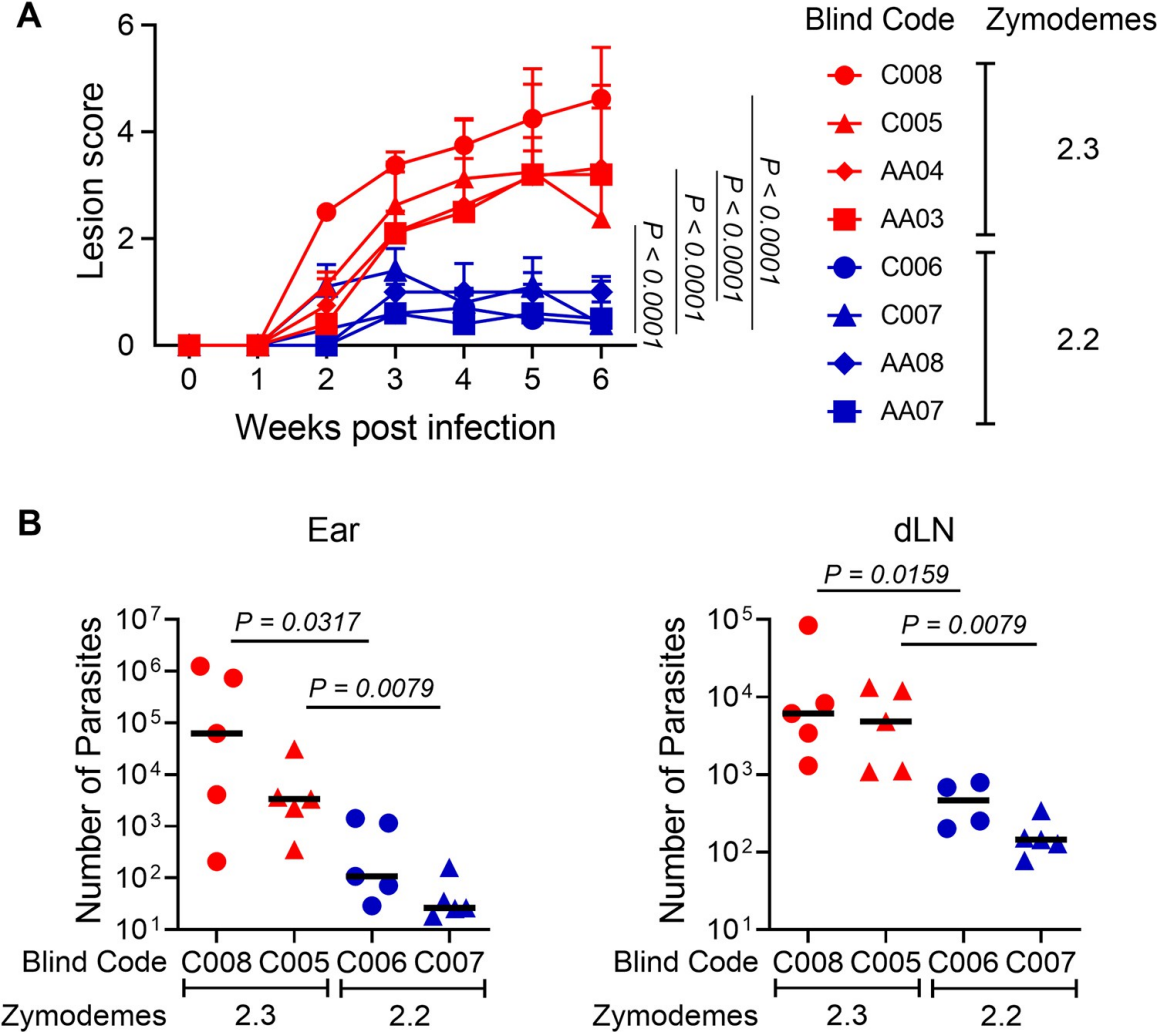
Pathogenicity of clinical strains of *L*. (*V*.) *panamensis* pertaining to Zymodemes 2.2 and 2.3 in the experimental BALB/c mouse model. BALB/c mice were infected intradermally in the ear with 10^5^ metacyclic parasites of *L*. (*V*.) *panamensis* strains pertaining to zymodeme 2.2 or zymodeme 2.3. **(A)** Lesion development over time was measured weekly, and lesion score determined based on the inflammation of the ear, lesion size (mm) and onset of necrosis in mice infected with 4 clinical strains of *L*. (*V*.) *panamensis* per zymodeme. [36]. **(B)** Parasite load at six weeks post-infection in the ear lesions and draining lymph nodes (dLNs) measured by limiting dilution assay (LDA). Data are representative of ≥ 2 independent experiments, each including Sb-sensitive zymodeme 2.2 (n = 2) and Sb-resistant zymodeme 2.3 (n = 2) strains. Strains analyzed within the same experiment are labeled with the same geometric symbol. Red and blue lines and symbols represent zymodemes 2.3 and 2.2, respectively, with error bars indicating standard error of the mean; n = 4 or 5 mice per strain. Lesion scores for individual strains were analyzed using two-way ANOVA considering two factors: time and strain. Parasite burden of strains corresponding to zymodeme 2.2 and 2.3 was compared using the Mann-Whitney test.

We evaluated the potential differences in pathogenicity by *L*. (*V*.) *panamensis* strains pertaining to zymodeme 2.2 and 2.3 using the experimental BALB/c mouse model. These *in vivo* studies demonstrated that BALB/c mice infected with zymodeme 2.3 strains developed significantly larger lesions; the difference becoming apparent between the 2^nd^ and 3^rd^-week post-infection and persisting throughout the 6-week observation period (Fig 6A). Additionally, the parasite burden at 6 weeks post infection was significantly higher in both the site of infection and the draining lymph node of mice infected with zymodeme 2.3 strains (Fig 6B).

## Discussion

This investigation sought to determine whether parasite susceptibility to meglumine antimoniate as measured *in vitro* or *ex vivo* was clinically relevant in the outcome of treatment. Importantly, prior clinical trials and prospective patient enrollment and monitoring provided access to clinical strains of *Leishmania* from patients having documentation of known risk factors for treatment failure, and the outcome of treatment for this integrated analysis of the interplay of host cell, parasite and antimonial drug in resistance and sensitivity to this anti-leishmanial drug. Additionally, the disparate susceptibility to meglumine antimoniate of subpopulations of *L*. (*V*.) *panamensis* distinguished by isoenzyme profiles, established that Sb resistance is a phenotype of biochemically discernable subpopulations of *L*. (*V*.) *panamensis*, rather than individual strains. The consistency of the disparate Sb susceptibility phenotypes of subpopulations of *L*. (*V*.) *panamensis* over more than four decades within the expansive geographic range of their transmission in Colombia reported by Fernández and collaborators [9] and confirmed in this study, substantiates the natural or primary nature of this resistance.

Establishing the relationship and relevance of *in vitro/ex vivo* resistance to the anti-leishmanial meglumine antimoniate and therapeutic response has been elusive [12–15]. Prior studies of antimonial drug susceptibility and clinical response to treatment have involved substantially smaller numbers of patients and strains [12,40], and have included multiple *Leishmania* species and diverse disease presentations such as mucosal involvement, which is long-known to be challenging to treat successfully. Dermal leishmaniasis caused by different *Leishmania* species has been shown to vary in clinical response to treatment with antimonial drugs [5, 7]. Therefore, the inclusion of patients infected with diverse *Leishmania* species could obscure the relationship with drug susceptibility.

The discovery of biochemically distinguishable naturally resistant and sensitive subpopulations of *L*. (*V*.) *panamensis* provided the opportunity to demonstrate that natural Sb-resistance is an important factor in the clinical response to treatment with meglumine antimoniate. The clinical relevance of the reported *in vitro/ex vivo* assessment of susceptibility of *L*. (*V*.) *panamensis* to meglumine antimoniate in human cutaneous leishmaniasis is corroborated by: 1) the significantly higher survival following in *vitro/ex vivo* exposure to the plasma C_max_ of SbV of strains isolated from patients who failed treatment; and 2) the significantly higher frequency of treatment failure among CL patients who were infected with naturally Sb-resistant zymodeme 2.3 strains compared to patients infected with the Sb sensitive zymodeme 2.2/2.1 strains. The calculated statistical powers of 99.6% achieved in determining the relationship between treatment outcomes and parasite susceptibility to antimonial drugs, and 87.5% for infections with naturally Sb-resistant and sensitive subpopulations distinguishable by zymodeme, support the analytical robustness of the study.

The early *in vivo* parasitological response to antimonial treatment at the lesion site also showed a modest yet significant difference in relation with infecting subpopulation in which zymodeme 2.3 strains presented lower *in situ* reduction of parasite burden in response to treatment with meglumine antimoniate, consistent with their higher survival during exposure to this drug *in vitro/ex vivo*. Notably, the pretreatment parasite burden in lesions of patients was significantly lower for those caused by strains of zymodeme 2.3 compared with zymodeme 2.2. This finding in patient lesions contrasts with the higher parasite burden of zymodeme 2.3 strains in the BALB/c mouse model during 6 weeks of infection, and likely reflects variation in behavior of *L*. (*V*.) *panamensis* infection in different host species. In particular, the general susceptibility of the BALB/c murine model to *Leishmania* infection including *L*. (*V*.) *panamensis* [35], together with the longer time of evolution of infection at the time of diagnosis and evaluation in patients, could influence parasite burden and the difference in experimental infection of BALB/c mice and patient lesions.

The identification of genetic markers of infections presenting distinct clinical manifestations such as disseminated cutaneous leishmaniasis caused by a population of *L*. (*V*.) *braziliensis* that is less responsive to meglumine antimoniate in Brazil, is consistent with the emergence of other naturally resistant subpopulations of different *Leishmania* species [41,42]. Interestingly, the aforementioned concurrence of poor response to meglumine antimoniate and pathogenicity was also observed in this investigation with clinical strains of the Sb-resistant zymodeme 2.3 subpopulation, which were more pathogenic in BALB/c mice than strains of the zymodeme 2.2 subpopulation.

The host response to infection with these subpopulations of *L*. (*V*.) *panamensis* is evidently interwoven with their responsiveness to antimonial drug. Infection of neutrophils with these subpopulations has been shown to differentially modulate various effector responses of this innate immune cell [43,44]. In line with the modulation of host cell response by drug resistant parasites, antimony resistant clinical strains of *L*. (*L*.) *donovani* were shown to induce the expression of host cell MRP-1 and P-gp, leading to active drug efflux [45]. The latter antimony resistant strains also inhibited leishmanicidal effects of sodium antimony gluconate and the induction of proinflammatory cytokines by this drug in dendritic cells [46]. Antimony resistant and sensitive clinical strains and laboratory derived lines of *L*. (*V*.) *panamensis* have also been found to distinctly modulate the expression of drug transporter genes involved in the response to antimony in human THP-1 macrophages and primary monocyte-derived macrophages [47]. The current findings substantiate the influence of natural *Leishmania* drug susceptibility phenotype on the host cell response to infection, as well as the antileishmanial potency of the drug.

Systematic evaluation of anti-leishmanial drug susceptibility of species across their respective geographic distribution has yet to be undertaken. Our findings and the reports of disparate therapeutic responses of other species of the *Viannia* subgenus to antimonial drug in different geographic regions of Brazil [42,48] and Argentina [49] suggest that intra-species variation is likely to be common and that tolerant or resistant subpopulations occur naturally and reflect intrinsic differences in the drug susceptibility, elicited host cell responses, and transmission epidemiology of distinct populations [50,51]. Characterization of the drug susceptibility of clinical strains isolated from patients residing in or originating from areas of transmission where effectiveness of standard-of-care treatment is poor, is likely to reveal other naturally tolerant or resistant subpopulations [41,42]. The detection of such naturally resistant subpopulations of *Leishmania* for antimonial and other anti-leishmanials provides the basis for definition of markers to identify these subpopulations, thereby informing epidemiologic risk and treatment guidelines, and potentially transforming prevention and control strategies.

While the disparate susceptibility of subpopulations of *L*. (*V*.) *panamensis* defined by zymodemes was clearly manifest and statistically significant in both the U937 macrophage *in vitro* and in primary macrophage and PBMC *ex vivo* models, meglumine antimoniate exhibited greater potency in primary human macrophages and PBMCs. Consequently, overall survival of clinical strains in primary host cells in the presence of meglumine antimoniate was lower for all of the subpopulations evaluated, and contributed to increased overlap in the proportional reduction of parasites of these phenotypically distinct subpopulations with respect to Sb susceptibility.

The intact innate immune functions and corresponding antimicrobial capacity of primary host cells compared with the changes induced by PMA (Phorbol myristate acetate) treatment or the loss or diminution of these functions in permanent cell lines such as U937 macrophages [52] may underlie this difference. Investigation of the host cell influence on the *in vitro* activity of antileishmanial drugs by Seifert and colleagues [53] similarly found that sodium stibogluconate displayed highest activity against *L*. (*L*.) *donovani* in monocyte-derived macrophages compared to primary mouse macrophages and THP-1 cells, presenting up to a 56-fold higher activity of this drug in monocyte-derived macrophages compared with differentiated THP-1 cells [53]. In the current study, the lower potency of meglumine antimoniate in U937 macrophages enabled the recognition of the natural Sb-resistance of the zymodeme 2.3 subpopulation of *L*. (*V*.) *panamensis* and ultimately, its association with treatment failure. These findings suggest that antimonial drug potency, and treatment failure are enabled by host responses elicited by phenotypically different parasite populations. The potency of other anti-leishmanials may likewise be influenced by host cell responses and merits consideration in their evaluation. The results of this study underscore the importance of the selection of the host cell for investigations of drug resistance, based on the question driving the research.

### Limitations

Biological assay of drug susceptibility of *Leishmania* presents challenges in relation with the arduous and costly methods of quantification of the viability of intracellular amastigotes and the inherent variability of biological systems, including different host cells and donors, and diverse geographic and patient contexts of clinical strains, as well as the extent of their propagation and passage following isolation. Despite these limitations, the evidence deriving from this analysis of 91 clinically documented CL patients in relation with the outcome of treatment with meglumine antimoniate and the Sb-susceptibility of the corresponding strains is consistent overall, and compelling. Genomic markers of the subpopulations defined by zymodeme classification that allow their identification by PCR have been ascertained and are being validated to facilitate diagnosis and effective treatment of CL caused by naturally Sb-resistant *Leishmania*. The underlying mechanisms and generalizability of the inter-relationship of parasite resistance and elicited host cell responses in the potency of other anti-leishmanials remain to be ascertained. Meanwhile, the results of this study document the importance of the interrelation between host and parasite factors in natural antimonial drug resistance.

Host factors that have been associated with an unfavorable outcome of treatment such as young or older age, co-morbid conditions, poor adherence to treatment, and lesions involving cartilaginous tissue are generally recognized as risk factors for failure of anti-leishmanial treatment [2,21–24]. The association of parasite Sb susceptibility and resistance *in vitro* with the clinical response to treatment with meglumine antimoniate was consistent and statistically significant both in the group of participants without risk factors for treatment failure and in the group that included 19/47 (40%) participants having one or more of the defined risk factors. Critically, and in contrast, this association was not evident when the 19 strains and corresponding patients having defined risk factors for, and effectively presented 68% treatment failure, were separately analyzed. Hence a high prevalence of risk factors for treatment failure among CL patients in particular settings and circumstances or studies, can, and likely has contributed to the inconsistent and previously uncertain association of parasite Sb resistance *in vitro*/*ex vivo* and treatment failure. These host-related risk factors can confound and obscure the relationship and should be taken into consideration in analyses of the association of anti-leishmanial drug resistance with treatment outcome.

### Conclusions

The results of this study establish the association of *Leishmania* Sb susceptibility *in vitro/ex vivo* with outcome of treatment, and the natural Sb-resistance of the subpopulation pertaining to zymodeme 2.3 with failure of antimonial drug treatment. Further, the findings demonstrate the clinical consequences of natural antimony resistance and the distinct pathogenicity of *L*. (*V*.) *panamensis* subpopulations distinguishable based on isoenzyme polymorphisms as Sb sensitive zymodemes 2.1 and 2.2, and Sb-resistant zymodeme 2.3. The higher antileishmanial potency of meglumine antimoniate in primary human macrophages and PBMCs compared to the *in vitro* susceptibility of intracellular amastigotes in U937 macrophages, is consistent with and suggests the participation of host cell responses elicited by parasite subpopulations corresponding with zymodemes 2.1/ 2.2, and zymodeme 2.3 in Sb susceptibility and the clinical response to antimonial drug [43]. These results support the clinical and epidemiological relevance of laboratory assessment of Sb susceptibility. Further, the findings underscore the importance of intrinsic characteristics of naturally occurring intraspecific heterogeneity of subpopulations of *Leishmania* in drug susceptibility and therapeutic response, and consideration of patient risk factors for treatment failure in evaluating this relationship.

## Supporting information

S1 Fig Standardization of drug exposure for evaluation of susceptibility in U937 macrophage model and quantitative readout of parasite survival using microcopy and qRT-PCR of *Leishmania* 7SLRNA. Evaluation of parasite burden by Microscopy **(A)** Previously established *in vitro* protocol for evaluation of susceptibility to antimonial drug exposure over 72 h with dosing at 24 and 72 h after infection and **(B)** Single dosing of antimony at 24 h. **(C)** Evaluation of susceptibility profile of subpopulations by qRT-PCR of 7SLRNA. **(D)** Correlation of qRT-PCR and microscopic quantification of parasite survival. **(E)** Antimony susceptibility (single dosing) of *L*. (*V*.) *panamensis* subpopulations prevalent in the Pacific Coast Region of Colombia from 1980 to 2022. Data are expressed as median % parasite survival at 32 μg SbV/mL, the maximum concentration (C_max_) of antimony in plasma during treatment with meglumine antimoniate [21]. Differences in drug susceptibility among 2.1, 2.2 and 2.3 zymodeme subpopulations of *L*. (*V*.) *panamensis* were determined using Kruskal-Wallis test and Dunn’s multiple comparisons test. Correlation between qRT-PCR and microscopy readout was determined by Spearman’s rank correlation coefficient. SbV: pentavalent antimony.
(TIF)

S1 Table Clinical characteristics of lesions. (DOCX)

S1 Data Dataset containing information for experimental human cell models (*ex vivo* and *in vitro*) and *in situ* data, and data from BALB/c mice. (XLS)
