# Supplementary figures and images for: Natural resistance to meglumine antimoniate is associated with treatment failure in cutaneous leishmaniasis caused by Leishmania (Viannia) panamensis
Source: PLoS Negl Trop Dis. 2024 May 6;18(5):e0012156. doi: 10.1371/journal.pntd.0012156 (PMC11098511; doi:10.1371/journal.pntd.0012156)

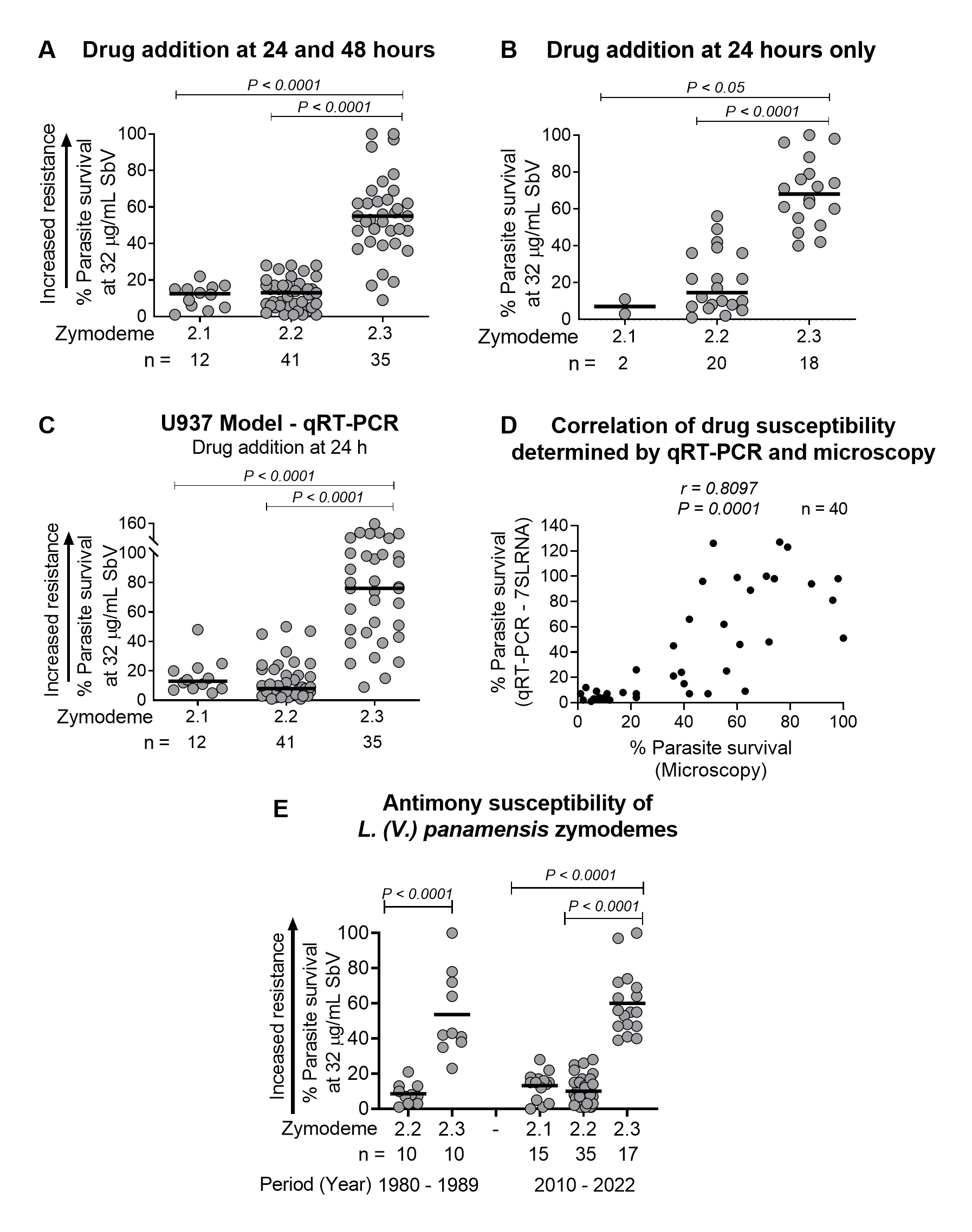

Supplement: S1 Fig — Evaluation of parasite burden by Microscopy (A) Previously established in vitro protocol for evaluation of susceptibility to antimonial drug exposure over 72 h with dosing at 24 and 72 h after infection and (B) Single dosing of antimony at 24 h. (C) Evaluation of susceptibility profile of subpopulations by qRT-PCR of 7SLRNA. (D) Correlation of qRT-PCR and microscopic quantification of parasite survival. (E) Antimony susceptibility (single dosing) of L. (V.) panamensis subpopulations prevalent in the Pacific Coast Region of Colombia from 1980 to 2022. Data are expressed as median % parasite survival at 32 μg SbV/mL, the maximum concentration (Cmax) of antimony in plasma during treatment with meglumine antimoniate [21]. Differences in drug susceptibility among 2.1, 2.2 and 2.3 zymodeme subpopulations of L. (V.) panamensis were determined using Kruskal-Wallis test and Dunn’s multiple comparisons test. Correlation between qRT-PCR and microscopy readout was determined by Spearman’s rank correlation coefficient. SbV: pentavalent antimony. (TIF) [file pntd.0012156.s001.tif]
