# Supplementary material for: Natural resistance to meglumine antimoniate is associated with treatment failure in cutaneous leishmaniasis caused by Leishmania (Viannia) panamensis
Source: PLoS Negl Trop Dis. 2024 May 6;18(5):e0012156. doi: 10.1371/journal.pntd.0012156 (PMC11098511; doi:10.1371/journal.pntd.0012156)
Supplement: S1 Table — (DOCX) [file pntd.0012156.s002.docx]

**S1 Table. Clinical characteristics of lesions**

| **Characteristic** | **Overall,** N = 91 | **Cure**, N = 52 | **Failure**, N = 39 | **p-value***^1^* |
| --- | --- | --- | --- | --- |
| Number of lesions, median (range) | 1 (1 - 9) | 1 (1 - 9) | 1 (1 - 6) | 0.4 |
| Number of lesions per patient, n (%): |  |  |  |  |
| One | 49 (54%) | 29 (56%) | 20 (51%) |  |
| Two | 22 (24%) | 13 (25%) | 9 (23%) |  |
| Three | 10 (11%) | 6 (12%) | 4 (10%) |  |
| Four or more | 10 (11%) | 4 (8%) | 6 (15%) |  |
| Type of lesion,  n (%) |  |  |  | 0.010 |
| Ulcer | 80 (88%) | 44 (55%) | 36 (45%) |  |
| Nodule | 1 (1%) |  | 1 (100%) |  |
| Papule | 1 (1%) |  | 1 (100%) |  |
| Plaque | 7 (8%) | 7 (100%) |  |  |
| Scar with active border | 1 (1%) | 1 (100%) |  |  |
| Other | 1 (1%) |  | 1 (100%) |  |
| Lesion area (mm)  Median (IQR, 25th–75th percentile) | 445.10 (188.25, 948.87) | 445.10  (231.20, 966.73) | 412.13 (137.38, 873.82) | 0.4 |
| *^1^* Fisher's exact test; Wilcoxon rank sum test | | | | |
